# Maximizing the Mini-Cog: Cognitive Decline Caused by Carotid Artery Stenosis

**DOI:** 10.26502/acmcr.96550217

**Published:** 2020-05-18

**Authors:** Shoshana Streiter, J. Michael Gaziano, Ariela R. Orkaby

**Affiliations:** 1 Division of Aging, Brigham and Women’s Hospital, Boston, MA, USA; 2 Geriatric Research, Education, and Clinical Center (GRECC), Massachusetts Veterans Epidemiology Research and Information Center (MAVERIC), VA Boston Healthcare System, Boston, MA, USA; 3 Division of Aging, Brigham & Women’s Hospital, Harvard Medical School, Boston, MA, USA

**Keywords:** Hypertension, Carotid artery stenosis, Diabetes, Geriatric

## Case Presentation

A 74-year-old man with a history of hypertension, hyperlipidemia, diabetes, and coronary artery disease presented for routine preventive care. His only concern was minimal word finding difficulty accompanied by occasional memory lapses that did not impair function. A Mini-Cog, a brief screening test for cognitive impairment, was performed. His result was abnormal and he was referred to Geriatrics.

On Geriatric evaluation he was independent in his activities of daily living, still working professionally, and managing his own finances and medications. Physical exam was unremarkable. Labs showed a normal TSH and RPR. Vitamin B12 was borderline low and he was started on oral supplementation. Brain MRI revealed non-specific white matter hyperintensities. On comprehensive neuropsychological testing he demonstrated average to above average performance across cognitive domains with mild deficits in short term recall and processing speed.

A month later he presented to the emergency department with acute confusion and worsening word finding difficulties. Symptoms resolved spontaneously within 20 minutes. CT angiography showed 95% left internal carotid artery stenosis; MRI showed new strokes in the left frontal and parietal lobes. He underwent a successful left carotid endarterectomy (CEA). One month later he reported complete resolution of his memory and word finding difficulties. Repeat neuropsychological testing showed superlative performance across all cognitive domains.

## 2. Discussion

Several screening tools for identification of cognitive impairment have been validated for clinical practice. One such tool is the Mini-Cog, a two-part test composed of a clock draw and delayed recall of three words. The test is considered positive if the patient either recalls 0/3 words or recalls 1–2 words and has an abnormal clock draw. The Mini-Cog takes approximately three minutes to perform and has been validated for use in the primary care setting [1].

Despite the availability of validated screening tools, the prevalence of undiagnosed cognitive impairment remains high. Barriers to early diagnosis include subtle presentation of disease, lack of provider confidence in making a diagnosis, and stigmatization of dementia [2]. Current recommendations from the United States Preventive Services Task Force (USPSTF) do not recommend for or against routine screening [3]. However, the clinician who makes an early diagnosis has the opportunity to identify and treat reversible etiologies, manage symptoms, and mobilize psychosocial supports for patients and families.

Cognitive decline has been recognized as a possible manifestation of carotid artery stenosis (CAS) and shown to improve following carotid revascularization [4]. Current guidelines recommend against screening for CAS in asymptomatic patients. The USPSTF defines symptoms as “TIA, stroke, or other neurologic signs or symptoms” [5]. It takes a high index of suspicion to identify subtle cognitive changes, recognize them as a symptom of CAS, and consider that the patient who manifests them may not represent the asymptomatic population in which screening is not recommended.

Our patient’s cognitive deficits resolved following a CEA. Moreover, concern about the effect of vascular disease on his cognition galvanized him into transforming his lifestyle. He modified his diet, started an exercise program, and lost over 40 pounds. Today, he remains active and independent, still working as a freelance photographer. His most recent clock draw and delayed recall were flawless.
